# Immunomodulatory role of *Emblica officinalis* in arsenic induced oxidative damage and apoptosis in thymocytes of mice

**DOI:** 10.1186/1472-6882-13-193

**Published:** 2013-07-27

**Authors:** Manish K Singh, Suraj S Yadav, Vineeta Gupta, Sanjay Khattri

**Affiliations:** 1 Department of Pharmacology, King George Medical University, Lucknow, Chowk 226 003, India

**Keywords:** Arsenic, *Emblica officinalis*, Oxidative stress, Apoptosis, Thymocytes

## Abstract

**Background:**

Arsenic is widely distributed in the environment and has been found to be associated with the various health related problems including skin lesions, cancer, cardiovascular and immunological disorders. The fruit extract of *Emblica officinalis* (amla) has been shown to have anti-oxidative and immunomodulatory properties. In view of increasing health risk of arsenic, the present study has been carried out to investigate the protective effect of amla against arsenic induced oxidative stress and apoptosis in thymocytes of mice.

**Methods:**

Mice were exposed to arsenic (sodium arsenite 3 mg/kg body weight p.o.) or amla (500 mg/kg body weight p.o.) or simultaneously with arsenic and amla for 28 days. The antioxidant enzyme assays were carried out using spectrophotometer and generation of ROS, apoptotic parameters, change in cell cycle were carried out using flow cytometer following the standard protocols.

**Results:**

Arsenic exposure to mice caused a significant increase in the lipid peroxidation, ROS production and decreased cell viability, levels of reduced glutathione, the activity of superoxide dismutase, catalase, cytochrome c oxidase and mitochondrial membrane potential in the thymus as compared to controls. Increased activity of caspase-3 linked with apoptosis assessed by the cell cycle analysis and annexin V/PI binding was also observed in mice exposed to arsenic as compared to controls. Co-treatment with arsenic and amla decreased the levels of lipid peroxidation, ROS production, activity of caspase-3, apoptosis and increased cell viability, levels of antioxidant enzymes, cytochrome c oxidase and mitochondrial membrane potential as compared to mice treated with arsenic alone.

**Conclusions:**

The results of the present study exhibits that arsenic induced oxidative stress and apoptosis significantly protected by co-treatment with amla that could be due to its strong antioxidant potential.

## Background

Arsenic is considered as an environmental contaminant and widely distributed in the environment due to its natural existence and anthropogenic applications [1,2]. It has long been used in pharmaceuticals, glass industries, manufacturing of sheep-dips, leather preservatives, poisonous baits, pesticides and semiconductor devices [3-5]. Exposure to arsenic in human could occur through air, soil and other occupational sources [6]. Human exposure to arsenic through contaminated food materials is quite common in the area having high levels of arsenic in ground water [6-8]. High levels of arsenic in ground water in India and many other regions of the world have been found to be associated with various health related problems including arsenicosis, skin lesions, cardiovascular diseases, reproductive problems, psychological, neurological and immunotoxic responses [2,9]. In view of increasing risk of arsenic toxicity, World Health Organization lowered the limit of arsenic in ground water from 50 μg/l to 10 μg/l [6].

Epidemiological studies have suggested that exposure to arsenic in humans may attributed to various immune related disorders [10-13]. In utero exposure to arsenic has been shown to suppress the immune mediated cells and impaired child thymic development associated with increased morbidity in children [14]. Exposure to arsenic during pregnancy has been found to lower thymic index suggested poor development of thymus in infants [15]. Increased placental inflammatory response, reduced placental T cells and altered levels of cord blood cytokines linked with fetal death, impaired infant health associated with enhanced oxidative stress have also been reported following exposure to arsenic [16,17]. Andrew et al. [10] observed that arsenic exposure may stimulate the over expression of genes involved in the defense system, immune function, cell growth, apoptosis, regulation of cell cycle, T cell receptor signaling pathway and diabetes. Association of arsenic intoxication with cancer, black foot disease and diabetes has also been reported [18,19]. Arsenic exposure in mice has been found to be associated with the increased free radical generation that affects the primary electron rich sites within the cells and cause DNA damage in human lymphocytes [20-22], breaking of DNA strand, DNA base modifications, protein crosslink, structural carbohydrates and lipids [23-25]. Arsenic induced cell death has been strongly linked to the induction of autophagy in human lymphoblastoid cell lines to impart its immunotoxic effects [11,12]. Numerous studies have reported that exposure to inorganic arsenic increased the frequency of micronuclei, chromosome aberrations and sister chromatid exchanges both in humans and experimental animals [26,27].

Recently, plant derived natural compounds and their active constituents have received great attention as a potential antioxidant against arsenic induced toxicity [21,28,29]. The fruit extract of *Emblica officinalis* (amla) with a history of medicinal value, long been used in Chinese and Indian traditional system of medicine and has shown anti-oxidative and immunomodulatory properties [30-33]. Amla contains a wide variety of phenolics including anthocyanins, flavonols, ellagic acid and its derivatives which protects against the harmful action of ROS and exhibits a wide range of biological effects including antioxidant, anti-tumour, anti-inflammatory, anti-bacterial and hepato-protective [32-34]. The dose of arsenic selected in the present study is quite low and based on the earlier studies [35,36]. Although a number of studies have been carried out to understand the protective efficacy of herbal agents against arsenic induced toxicity, not much is known about its mechanism involved in immunotoxicity and protective management. In recent years, the intake of dietary polyphenols has received a great attention of health scientists to use them in the therapeutic management of various disease conditions. The present study has therefore been focused to investigate the immunomodulatory role of the fruit extract of amla in arsenic induced oxidative damage including lipid peroxidation, status of antioxidant enzymes and mitochondrial membrane potential and apoptosis and necrosis in thymocytes of mice.

## Methods

### Chemicals

Sodium arsenite, RNase A, 2′,7′-dichlorofluorescein diacetate (DCFH-DA), 3-(4,5-dimethyl-2-yl)- 2,5-diphenyl tetrazolium bromide (MTT), 7-amino-4-trifluoro methylcoumarin (AFC), DPPH (1,1-diphenyl-2-2′-picrylhydrayl) and all other chemicals were purchased from Sigma–Aldrich, USA. Rhodamine 123 (Rh 123) and from Molecular Probes, propidium iodide (PI) from Calbiochem, and Annexin V-FITC were purchased from Biovision.

### Plant material

Studies have been reported that phenolics and flavonoids including gallic acid, ellagic acid, isocorilagin, chebulanin and chebulagic acid are the major constituents present in ethyl acetate extract of amla [37,38]. To investigate the combined effect of theses constituents, ethyl acetate extract of amla has been selected for the present study. Briefly, the fresh fruits of amla were collected from authentic source and fruit powder was extracted three times using 95% ethanol (Plant Identification No. - 13394 obtained by the Herbarium of Birbal Sahni Institute of Palaeobotany, Lucknow, India). The combined extracts were filtered and evaporated to dryness with a rotary evaporator under reduced pressure and the residue was suspended in water and extracted successively with diethyl ether and ethyl acetate. The extract was evaporated under reduced pressure to get powdered form of ethyl acetate fraction.

### Animals and treatment

The Balb/c male mice (15 ± 2 g) were obtained from the animal breeding colony of CSIR-Indian Institute of Toxicology Research, Lucknow used for the study. Mice were housed in an air-conditioned room at 25 ± 2°C with a 12 h light/dark cycle under standard hygiene conditions and had free access to pellet diet and water *ad libitum*. The study was approved by the institutional animal ethics committee of King George Medical University, Lucknow (No. 121 IAH/Pharma-11) and all experiments were carried out in accordance with the guidelines laid down by the committee for the purpose of control and supervision of experiments on animals (CPCSEA), Ministry of Environment and Forests (Government of India), New Delhi, India. The animals were randomly divided into four groups contained ten animals in each group as follows

Group I. Mice were treated with vehicle (2% gum acacia) for the duration of the treatment to serve as controls.

Group II. Mice were treated with arsenic as sodium arsenite (dissolved in distilled water 3 mg/kg body weight p.o., daily for 30 days).

Group III. Mice were treated with fruit extract of amla (500 mg/kg body weight, suspended in 2% gum acacia, p.o., daily for 30 days).

Group IV. Mice were co-treated with arsenic and fruit extract of amla identically as in group II and III.

After the last dose of treatment, animals were sacrificed and thymus of all the animals was isolated. Out of all isolated thymus three of each set, put into the phosphate buffer saline (PBS) and processed for the measurement of apoptotic parameters. While remaining thymus were immediately placed in an ice cold saline solution (0.15M), blotted on filter paper, quickly weighed and processed for enzymatic and non-enzymatic antioxidants assays.

### Preparation of thymocyte suspension

Thymus was dissected from mice and single cell suspension was prepared under aseptic condition. The suspension was passed through stainless steel mesh centrifuged at 200 × g at 4°C for 10 min and resuspended in complete cell culture medium (RPMI-1640 containing HEPES and 2mM glutamine, supplemented with 10% FBS and 1% antibiotic antimycotic solution) the cell density were adjusted to 1.5 × 10^6^ cell/ml.

### Measurement of antioxidant activity

#### Assay of DPPH radical scavenging activity

The free radical scavenging activity of ethyl acetate extract amla was measured by the scavenging of the DPPH radical using the standard method with slight modifications [39]. Amla extract at different concentrations (1.0, 2.5, 5, 10, 20 and 40 μg/ml) in ethanol (2ml) was mixed with DPPH (2 ml, 1mM in ethanol) and incubated for 30 min in the dark. The absorbance of DPPH radical was read at 517 nm using a spectrophotometer. The DPPH radical scavenging activity was calculated using the following equation

$$\mathrm{Scavenging}\;\mathrm{activity}\;\left(\%\right)=\left\{\left(A_{0}-A_{1}\right)/A_{0}\right\}\times 100$$

where *A*_0_ is the absorbance of the control reactions and *A*_1_ is the absorbance in the presence of the test compound.

### Biochemical parameters

#### Assessment of cell viability

The cell viability was measured by the MTT reduction method following the standard procedure [40]. Cells were seeded at a density of 1.0 × 10^4^ in a 96 well plate. 10 μl of MTT (5 mg/ml PBS) was added to the each well and incubated for 4 h at 37°C in a CO_2_ incubator. The plate was centrifuged at 1200 × g for 10 min and 100 μl of DMSO was added after aspirating supernatant to dissolve the formazan formed in the wells. After 5 min the absorbance was read on a microplate reader (Synergy HT of BIO-TEK International, USA) at 530 nm.

#### Assay of lipid peroxidation

Lipid peroxidation as a measure of thiobarbituric acid reactive substances was measured following the standard procedure [41]. Malondialdehyde (MDA) forms as an intermediate product of the peroxidation of lipids and serves as an index of the intensity of oxidative stress. The intensity of pink color formed during the reaction was read on a spectrophotometer at 532 nm.

#### Assay of reduced glutathione levels

Levels of reduced glutathione (GSH) in the thymus homogenate were estimated following the standard protocol [42]. The assay involves the reaction of GSH with 5, 5′-dithiobis-2 nitrobenzoic acid (DTNB) that forms yellow color and its absorbance was taken by spectrophotometer at 412 nm. The result has been expressed at μg GSH/mg protein.

#### Assay of superoxide dismutase activity

Activity of superoxide dismutase in thymus homogenate was assayed according to the method of Marklund and Marklund [43]. The unit of enzyme activity is defined as the enzyme required for 50% inhibition of pyrogallol auto-oxidation. The results have been expressed as unit/min/mg protein.

#### Assay of catalase activity

Activity of catalase in thymus homogenate was assayed following the standard protocol [44] using hydrogen peroxide (H_2_O_2_) as substrate. The activity of catalase was measured on a spectrophotometer and has been expressed in μmole/min/mg protein.

#### Assay of caspase-3 activity

Activity of caspase-3 in thymocytes was measured following the standard procedure described by Pathak and Khandelwal [45]. Briefly, the cells (3.0 × 10^6^/ml) were lysed on ice for 10 min with the help of lysis buffer. Further, the reaction buffer (10 mM Tris–HCl, 1 mM EDTA, 10 mM DTT, 5% glycerol) and DEVD-AFC substrate (50 μM) were added and incubated at 37°C in dark for 2 h. AFC was used as standard and fluorescence was measured at excitation and emission wavelengths of 400 nm/505 nm, respectively, on a microplate reader. The enzyme activity is expressed as nmoles AFC/60 min.

#### Assay of cytochrome c oxidase activity

Cytochrome c oxidase activity was assayed through the colorimetric assay kit purchased from Sigma-Aldrich Chemical Co. (St. Louis, MO, USA). Absorbance was measured on a spectrophotometer at 550 nm and values are expressed as U/ml.

#### Assay of ROS generation

The generation of ROS was measured using DCFH-DA by flow cytometry as described previously [46]. Single cell suspension (1 × 10^6^/ml) of thymus from control and treated mice was suspended in PBS and incubated with DCFH-DA at 37°C for 1 h. Hydrolysis of DCFH-DA leads to formation of fluorescence DCFH that was measured by the fluorescence intensity (FL-1, 530 nm).

#### Assay of mitochondrial membrane potential

The detection of mitochondrial membrane potential was assessed by flow cytometry following the standard procedure [47]. Single cell suspension (1 × 10^6^/ml) was incubated with Rh-123 for 60 min in dark at 37°C. The mitochondrial membrane potential was measured using FL-1 filter at fluorescence intensity of 530 nm.

### Analysis of apoptotic DNA

The assay of apoptotic DNA was carried out using the standard procedure [48]. Briefly, cell suspension of thymocytes at a density of 1 × 10^6^ cells from control and treated mice was prepared for the detection of cell cycle analysis. Cells were washed with PBS and fixed by 70% ethanol. The fixed cells were again washed with PBS and added phosphate citrate buffer (200 μl, pH 7.8) and incubated for 60 min at room temperature. After centrifugation the cells were resuspended in 0.5 ml propidium iodide (PI) and 0.5 ml RNAse (50 μg/ml) and further incubated for 30 min in the dark. The PI fluorescence was measured through a FL-2 filter (585 nm) and a total of 10,000 events were acquired.

### Assessment of apoptotic and necrotic cell

The apoptotic and necrotic cell distribution was analyzed through Annexin V binding and PI uptake following the procedure of Vermes et al. [49]. Briefly, thymocytes were suspended in 1 ml binding buffer (1×), an aliquot of 100 μl was incubated with 5 μl Annexin V-FITC and 10 μl PI for 15 min in dark at room temperature and 400 μl binding buffer (1×) was added to each sample, the FITC and PI fluorescence will be measured through FL-1 (530 nm) and FL-2 filters (585 nm) respectively.

### Protein estimation

Protein concentration in thymus homogenates was measured following the standard procedure [50] using bovine serum albumin as the reference standard.

### Statistical analysis

The statistical analysis was carried out by GraphPad Prism 3.02 using one way analysis of variance followed by Newman–Keuls test for multiple pair wise comparisons among the groups. All values have been expressed as mean ± SEM. P value <0.05 has been considered significant.

## Results

### Effect on DPPH free radical scavenging activity

**Figure 1:**
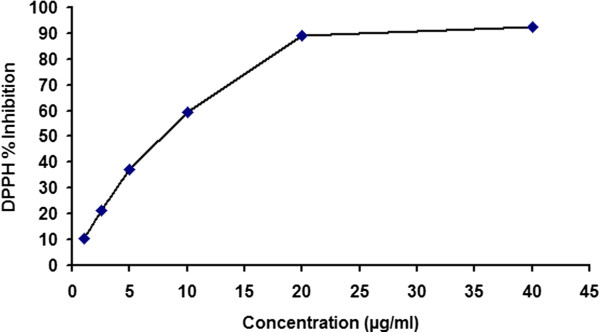
**DPPH free radical scavenging activity of fruit extract of *Emblica officinalis.*** The concentration of extract above 20 μg/ml showed a saturation in the plot indicating the radical scavenging activity of more than 90%.

The different concentration of ethyl acetate extract of amla (1.0, 2.5, 5 10, 20 and 40 μg/ml) in ethanol (2 ml) was used for the DPPH radical scavenging activity and results were presented in Figure 1. The 50% inhibitory concentration (IC_50_) of fruit extract was found to be 8.32 μg/ml and it become saturated over 20 μg/ml concentration where the activity was more than 90%. The results showed that ethyl acetate extract of amla has strong free radical scavenging activity associated with its antioxidant potential.

### Effect on body weight and thymus weight in mice

Effect of arsenic and co-treatment of arsenic and amla on mice has been presented in Table 1. Exposure to arsenic in mice caused a significant decrease in body weight (25%, p < 0.01) and thymus weight (34%, p < 0.001) as compared to controls suggesting the general toxic effect of the arsenic and could be associated with decreased food consumption and water intake. Co-treatment with arsenic and amla increased the body weight (21%, p < 0.05) and thymus weight (26%, p < 0.05) as compared to mice treated with arsenic alone. No significant effect on body weight and thymus weight was observed in mice treated with amla alone as compared to controls (Table 1).

**Table 1:** Effect on body, thymus weight and thymus cellularity in mice exposed to arsenic, amla and their co-treatment for 30 days

**Parameters**	**Control**	**Treatment groups**		
		**Arsenic**	**Amla**	**Amla**
		**(3 mg/kg)**	**(500 mg/kg)**	**+ Arsenic**
**Body weight (g)**	19.40 ± 1.12	15.40 ± 0.97^*a^	19.80 ± 0.80	18.60 ± 1.16^*b^
**Thymus weight (mg)**	79.2 ± 3.73	52.2 ± 3.26^*a^	77.6 ± 3.29	66.2 ± 4.54^*b^
**Thymus cellularity (×10)**^**-6**^	32.3 ± 6.60	26.2 ± 5.30^*a^	33.1 ± 5.20	30.6 ± 4.80^*b^

Values are mean ± SEM of five animals in each group.

*a-compared to control group; *b-compared to arsenic treated group.

*Significantly differs (p < 0.05).

### Effect on cell viability in thymus of mice

**Figure 2:**
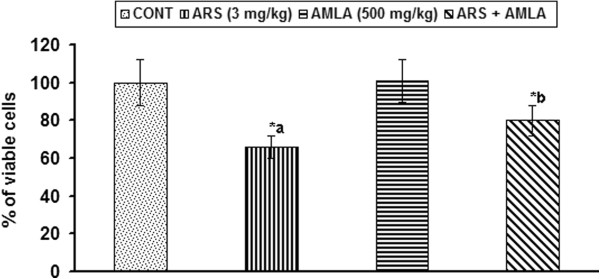
**Effect of arsenic, amia and their co-treatment on cell viability in thymus of mice.** Values are mean ± SEM of five animals in each group a-compared to control group, b-compared to arsenic treated group *Significantly differs (p < 0.05).

Effect of arsenic and co-treatment of arsenic and amla on cell viability in thymus has been presented in Figure 2. Mice exposed to arsenic exhibited a significant decrease in cell viability (34%, p < 0.001) as compared to controls. Co-treatment with arsenic and amla increased the cell viability (21%, p < 0.01) in thymus as compared to those treated with arsenic alone. No significant effect on cell viability was observed in mice treated with amla alone as compared to controls (Figure 2).

### Effect on the lipid peroxidation in thymus of mice

**Figure 3:**
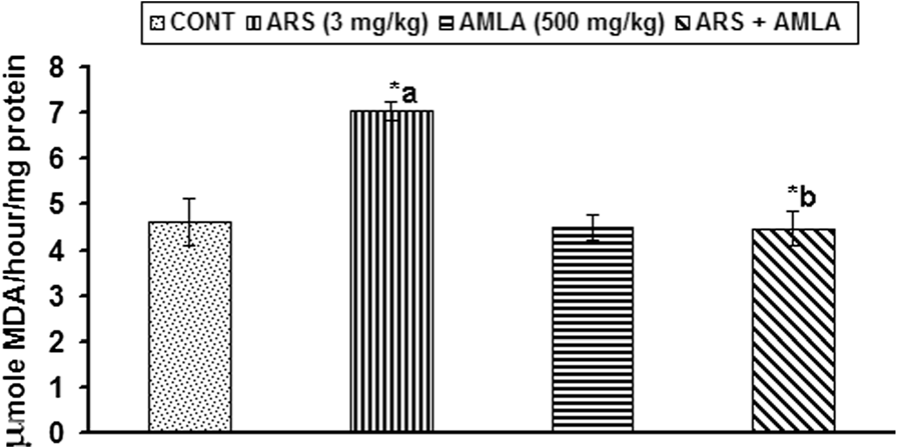
**Effect of arsenic, amla and their co-treatment on the levels of lipid peroxidation in thymus of mice.** Values are mean ± SEM of five animals in each group a-compared to control group, b-compared to arsenic treated group *Significantly differs (p < 0.05).

To assess the level of oxidative damage to the biological membrane, effect of arsenic and co-treatment of arsenic and amla on lipid peroxidation in thymus has been carried out and presented in Figure 3. A significant increase in lipid peroxidation (52%, p < 0.001) in thymus was observed in mice exposed to arsenic as compared to controls. Co-treatment with arsenic and amla decreased in the level of lipid peroxidation (36%, p < 0.001) in thymus as compared to those treated with arsenic alone. No significant effect on the level of lipid peroxidation was observed in mice treated with amla alone as compared to controls (Figure 3).

### Effect on the reduced glutathione levels in thymus of mice

**Figure 4:**
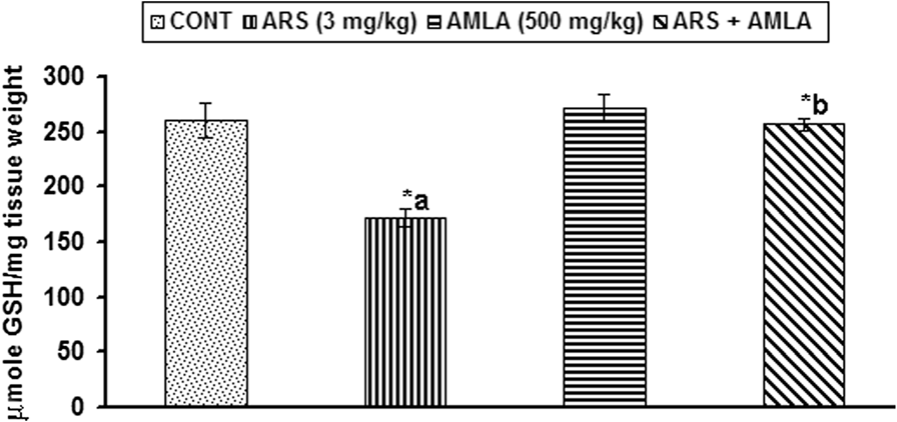
**Effect of arsenic, amla and their co-treatment on the levels of reduced glutathione levels in thymus of mice.** Values are mean ± SEM of five animals in each group a-compared to control group, b-compared to arsenic treated group *Significantly differs (p < 0.05).

Arsenic has high affinity to GSH and thus enhances vulnerability towards oxidative stress. Figure 4 indicates the effect of arsenic and co-treatment of arsenic and amla on reduced glutathione levels in thymus of mice. Exposure to arsenic in mice caused a significantly decreased in the levels of reduced glutathione (34%, p < 0.001) in thymus as compared to controls. Co-treatment with arsenic and amla increase the levels of reduced glutathione (49%, p < 0.001) in thymus of mice as compared to those treated with arsenic alone. No significant effect on the levels of reduced glutathione was observed in the thymus of mice treated with amla alone as compared to controls (Figure 4).

### Effect on the activity of superoxide dismutase in thymus of mice

**Figure 5:**
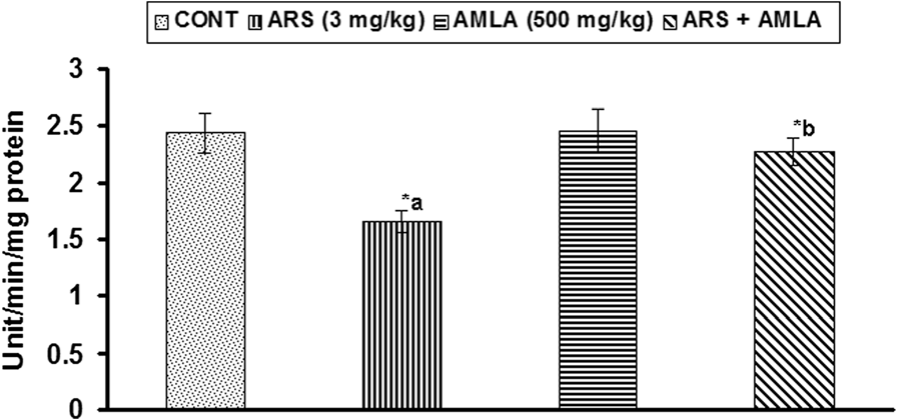
**Effect of arsenic, amla and their co-treatment on the activity of superoxide dismutase in thymus of mice.** Values are mean ± SEM of five animals in each group a-compared to control group, b-compared to arsenic treated group *Significantly differs (p < 0.05).

Effect of arsenic and co-treatment of arsenic and amla on the activity of superoxide dismutase in thymus has been presented in Figure 5. The activity of superoxide dismutase, an enzyme involved in the dismutation of superoxide radicals, was found to be significant decreased (31%, p < 0.01) in thymus of mice exposed to arsenic as compared to controls. Co-treatment with arsenic and amla increased the activity of superoxide dismutase (36%, p < 0.05) in thymus as compared to those treated with arsenic alone. No significant effect on the activity of superoxide dismutase was observed in the thymus of mice treated with amla alone as compared to controls (Figure 5).

### Effect on the activity of catalase in thymus of mice

**Figure 6:**
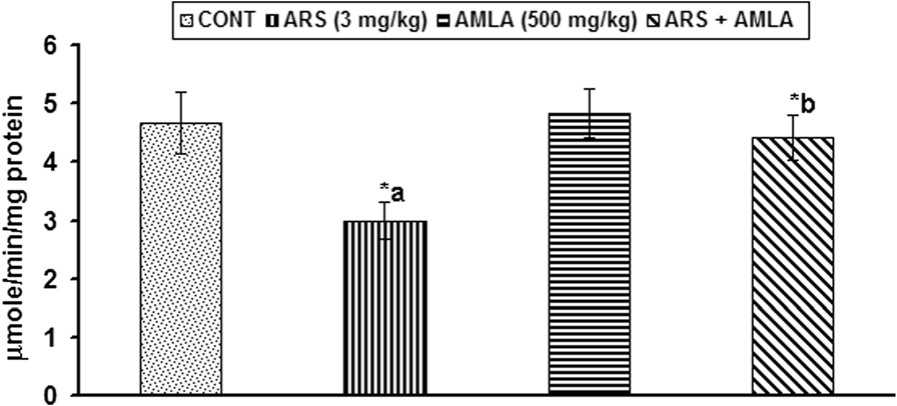
**Effect of arsenic, amla and their co-treatment on the catalase activity in thymus of mice.** Values are mean ± SEM of five animals in each group a-compared to control group, b-compared to arsenic treated group *Significantly differs (p < 0.05).

Effect of arsenic and co-treatment of arsenic and amla on the activity of catalase in thymus has been presented in Figure 6. Exposure to arsenic in mice caused a significant decrease the activity of catalase (35%, p < 0.05) in thymus of mice as compared to controls. Co-treatment with arsenic and amla increased the activity of catalase in thymus (32%, p < 0.05) as compared to those treated with arsenic alone suggesting a protective effect of amla against oxidative insult. No significant effect on the activity of catalase was observed in the thymus of mice treated with amla alone as compared to controls (Figure 6).

### Effect on caspase-3 activity in thymus of mice

**Figure 7:**
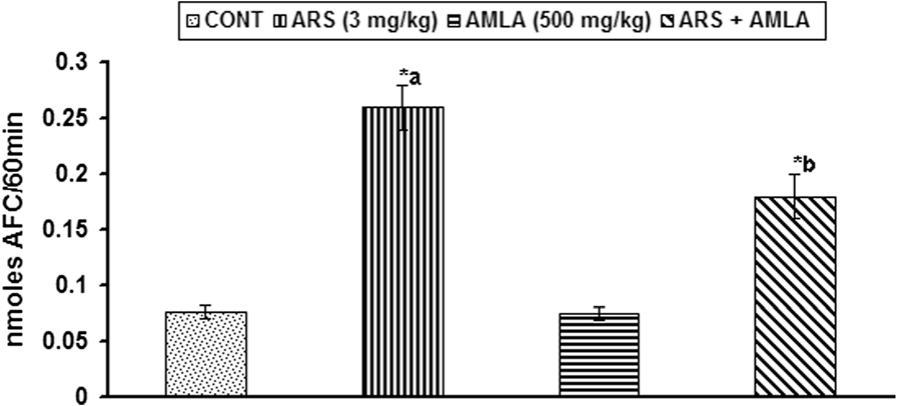
**Effect of arsenic, amla and their co-treatment on the catalase-3 activity in thymus of mice.** Values are mean ± SEM of five animals in each group a-compared to control group, b-compared to arsenic treated group *Significantly differs (p < 0.05).

Figure 7 demonstrate the effect of arsenic and co-treatment of arsenic and amla on the activity of caspase-3 in thymus of mice. Exposure to arsenic caused a significant increased in the caspase activity (2.42 fold, p < 0.001) in thymus of mice as compared to controls. Co-treatment with arsenic and amla decrease the activity of caspase (0.3 fold, p < 0.01) in thymus as compared to mice treated with arsenic alone suggesting a trend of recovery. No significant effect on the caspase activity was observed in mice treated with amla alone as compared to controls (Figure 7).

### Effect on cytochrome c oxidase activity in thymus of mice

**Figure 8:**
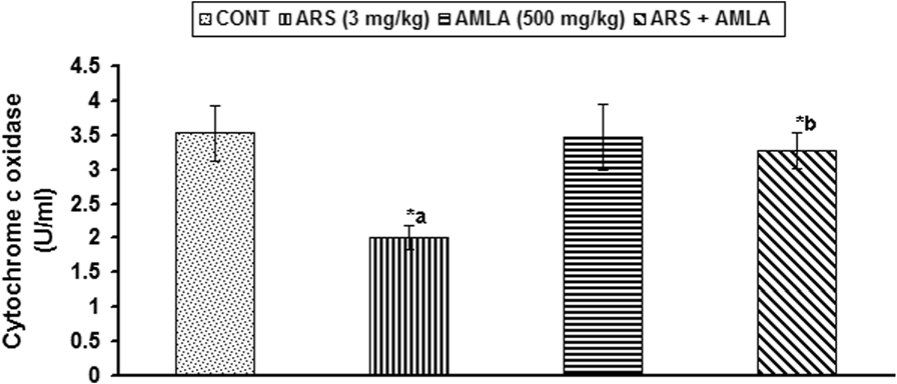
**Effect of arsenic, amla and their co-treatment on the cytochrome c oxidase activity in thymus of mice.** Values are mean ± SEM of five animals in each group a-compared to control group, b-compared to arsenic treated group *Significantly differs (p < 0.05).

Effect of arsenic and co-treatment of arsenic and amla on the cytochrome c oxidase activity in thymus has been presented in Figure 8. Exposure of arsenic in mice showed a decreased cytochrome c oxidase activity (43%, p < 0.001) in thymus as compared to controls. Co-treatment with arsenic and amla increased the cytochrome c oxidase activity (62%, p < 0.001) in thymus as compared to mice treated with arsenic alone. No significant effect on the cytochrome c oxidase activity was observed in mice treated with amla alone as compared to controls (Figure 8).

### Effect on the generation of ROS in thymus of mice

**Figure 9:**
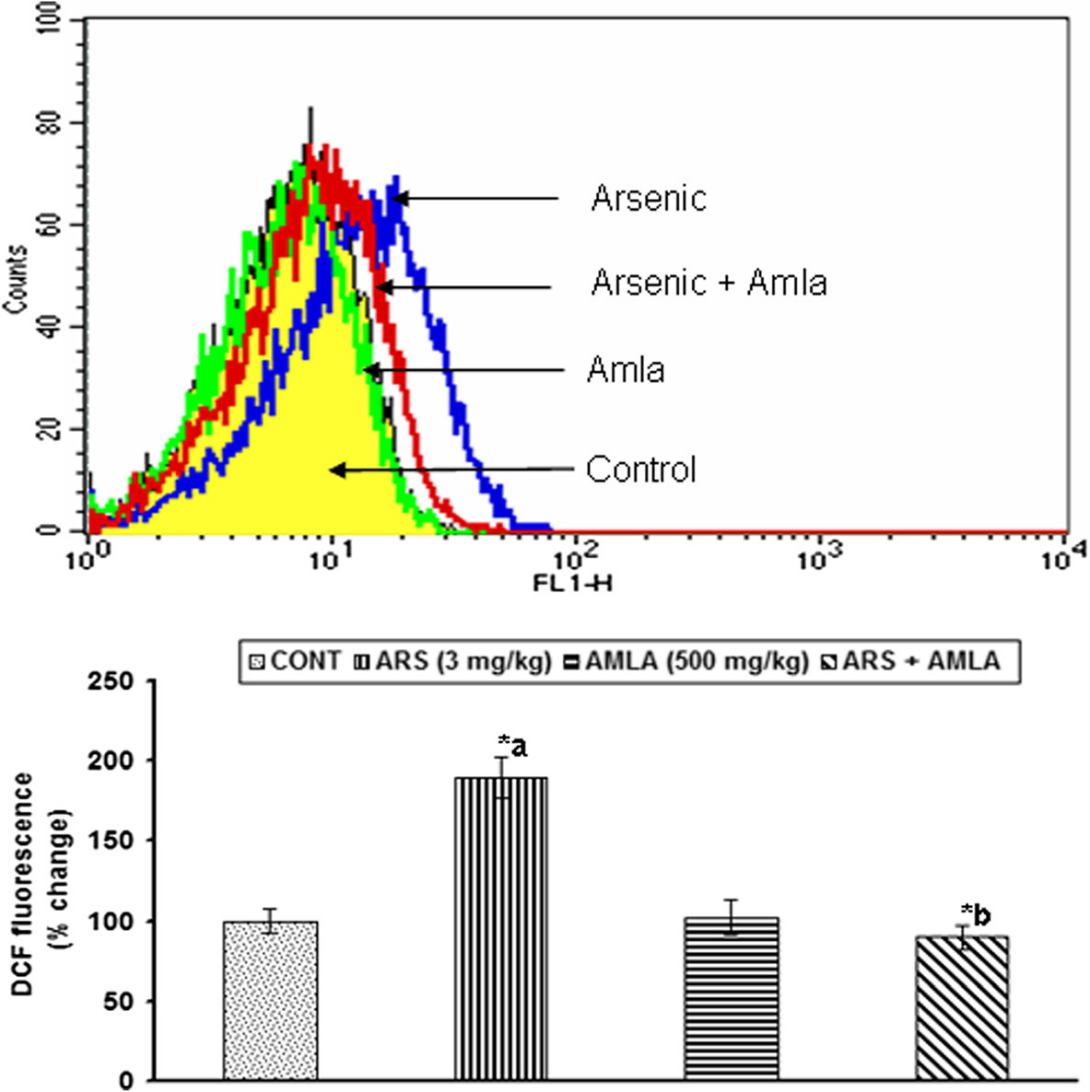
**Effect of arsenic, amla and their co-treatment on the generation of reactive oxygen species in thymocytes of mice.** Cells were incubated with DCFH-DA and fluorescence was measured using a flow cytometer with FL-1 filter. Values are presented as mean ± SEM of three assays performed independently a-compared to control group, b-compared to arsenic treated group *Significantly differs (p < 0.05).

Arsenic has been found to be associated with the increased generation of ROS. Effect of arsenic and co-treatment of arsenic and amla on ROS generation in thymus has been presented in Figure 9. Exposure of arsenic to mice caused an increased generation of ROS (90%, p < 0.01) in thymus as compared to controls. Co-treatment with arsenic and amla decreased the ROS generation (52%, p < 0.01) in thymus as compared to mice treated with arsenic alone suggested the antioxidant and free radical scavenging activity of amla. No significant effect on the production of ROS was observed in mice treated with amla alone as compared to controls (Figure 9).

### Effect on the mitochondrial membrane depolarization in thymus of mice

**Figure 10:**
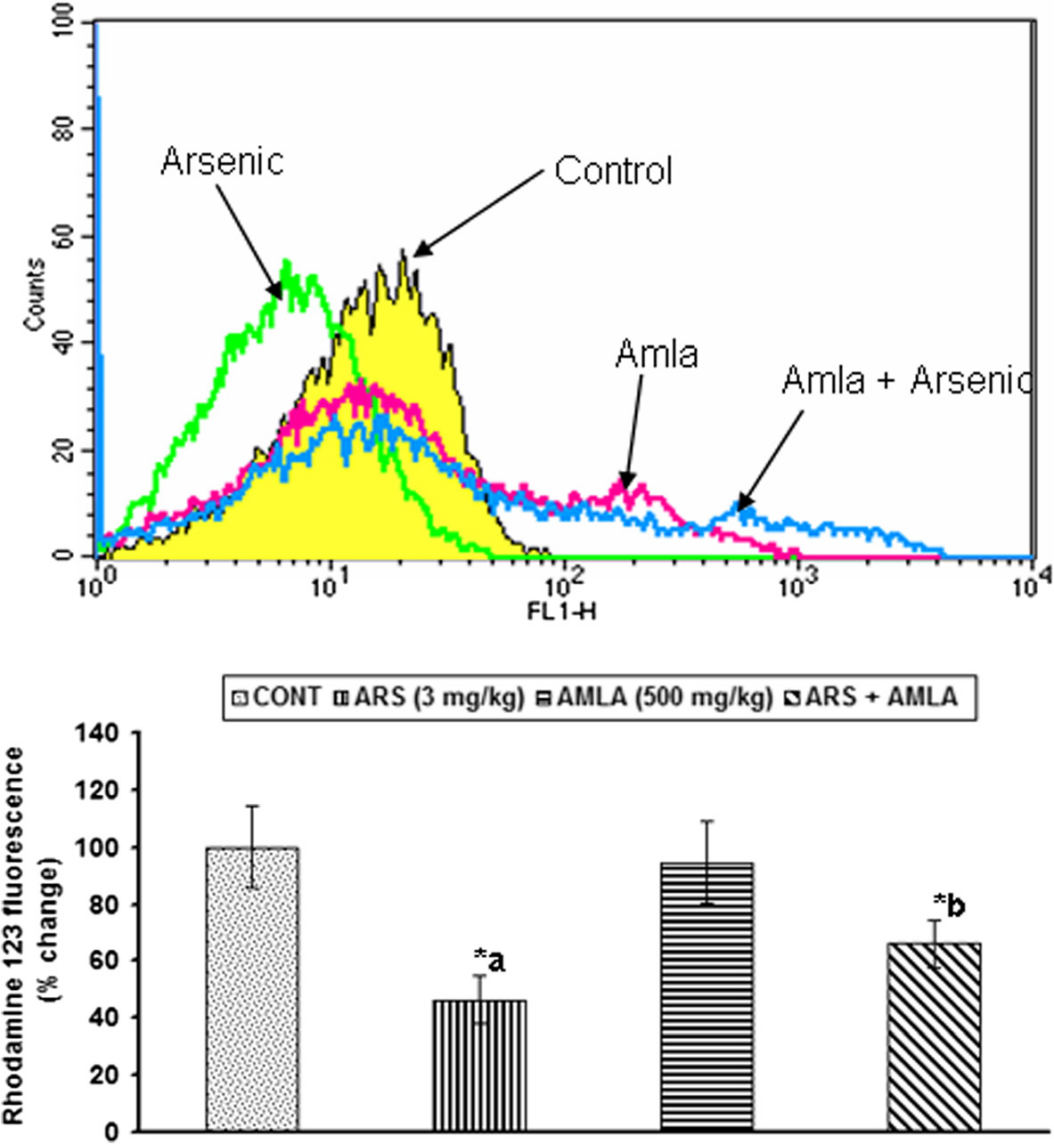
**Effect of arsenic, amla and their co-treatment on mitochondrial membrane potential of thymocytes of mice.** Cells were incubated with Rh 123 and fluorescence was measured using flow cytometer with FL-1 filter. Values are presented as mean ± SEM of three assays performed independently a-compared to control group, b-compared to arsenic treated group *Significantly differs (p < 0.05).

Effect of arsenic and co-treatment of arsenic and amla on mitochondrial membrane depolarization in thymus has been presented in Figure 10. Exposure of arsenic in mice showed decrease mitochondrial membrane depolarization (53%, p < 0.001) in thymus as compared to controls. Co-treatment with arsenic and amla increased the mitochondrial membrane depolarization (43%, p < 0.05) in thymus as compared to mice treated with arsenic alone. No significant effect on the mitochondrial membrane depolarization was observed in mice treated with amla alone as compared to controls (Figure 10).

### Effect on the cell cycle in thymus of mice

**Figure 11:**
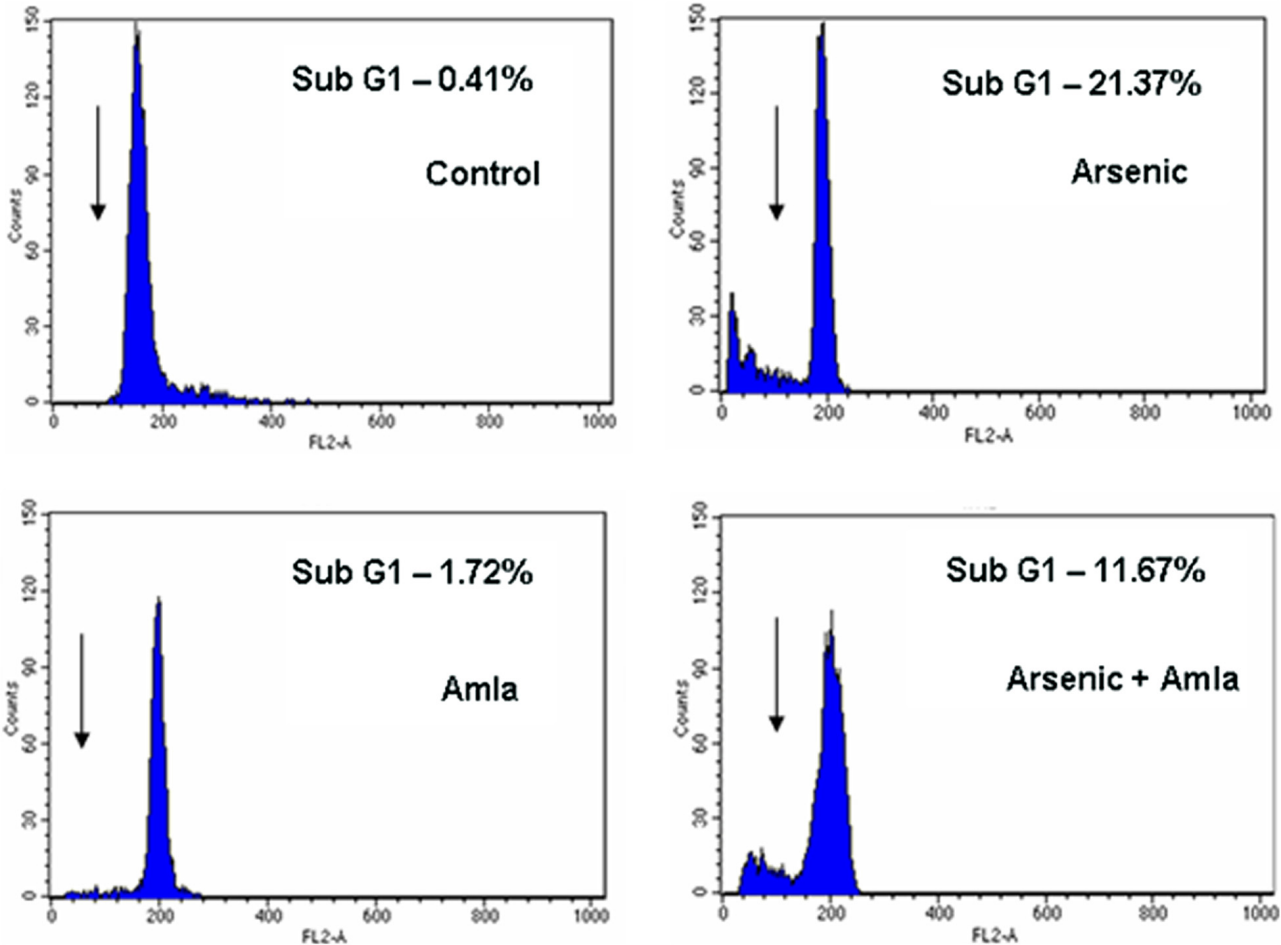
**Effect of arsenic, amla and their co-treatment on the cell cycle progression of thymocytes of mice.** Control and amla treated cells showed no sub-G1 peak while arsenic showed high numbers of cells in sub-G1 peak and group co-treated with of arsenic and amla showed reduce number of cells in sub-G1 peaks as compared to arsenic. Cells were incubated with propidium iodide and flourescence was measured using flow cutometer with FL-2 filter. Values are presented in the historam as mean ± SEM of three assays performed independently representing sub-G1 population of cells.

Cell cycle represents the DNA damage in cells in sub G1 peak. Figure 11 demonstrate the effect of arsenic and co-treatment of arsenic and amla on the DNA damage as observed by a sub G1 peak in thymus. Exposure of arsenic to mice caused an increased number of sub G1 peak indicating the DNA damage (21.37%, p < 0.01) in thymus as compared to controls. Co-treatment with arsenic and amla reduced the number of cells in sub G1 peak (11.67%, p < 0.01) in thymus as compared to mice treated with arsenic alone. No significant effect on the cell cycle was observed in mice treated with amla alone as compared to controls (Figure 11).

### Effect on the annexin V/PI binding assay in thymus of mice

**Figure 12:**
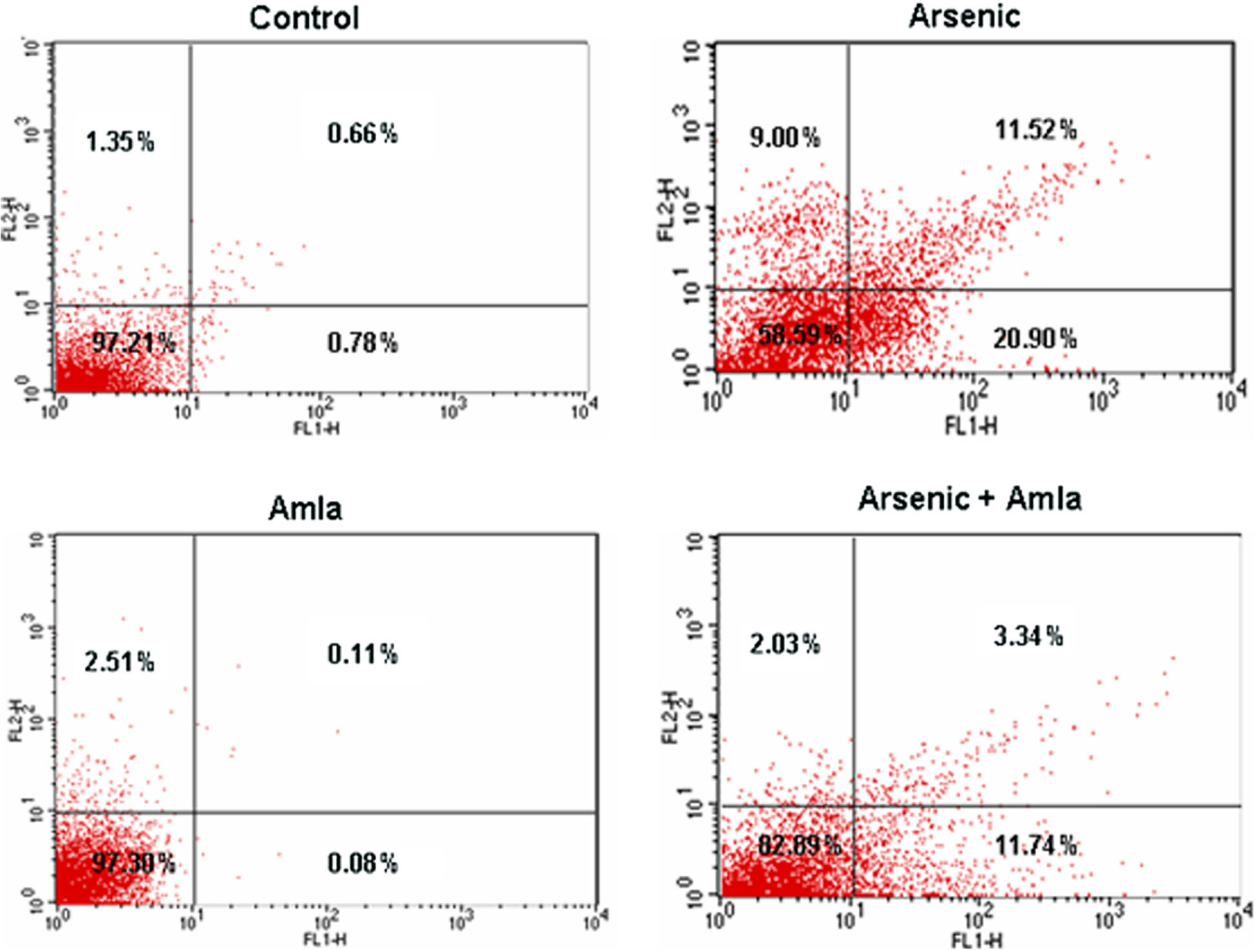
**Effect of arsenic, amla and their co-treatment on the annexin-V/PI dual staining of thymocytes of mice.** Cells in the upper right (UR) portion are late apoptotic cells, upper left (UL) are necrotic cells whereas cells in the lower left (LL) and lower right (LR) portion are viable and early apoptotic cells respectively. Values are presented as mean ± SEM of three assays performed independently.

Annexin binding assay has been used to measure the number of apoptosis and necrotic cells. Effect of arsenic and co-treatment of arsenic and amla on the apoptosis in thymus has been presented in Figure 12. Exposure of arsenic in mice caused an increased number of necrotic (9%) and apoptotic cells (11.52%) in thymus as compared to controls. Co-treatment with arsenic and amla decreased the number of necrotic (2.03%) and apoptotic cells (3.34%) in thymus as compared to mice treated with arsenic alone. No significant effect of apoptosis and necrosis in cells of the thymus was observed in mice treated with amla alone as compared to controls (Figure 12).

## Discussion

Enhanced oxidative stress has been found to play a crucial role in the induction of apoptosis under both pathological and physiological conditions. Increased generation of free radicals associated with enhanced oxidative stress has been found to be implicated in arsenic induced thymic atrophy [20,21]. Numerous studies have reported that exposure to arsenic increased the production of free radical generation and cause oxidative damage to the biological membrane through increased levels of lipid peroxidation, protein carbonyl contents followed by decreased antioxidant defense system [20,21,28]. GSH is an important biomolecule involve in the antioxidant defense system against toxicants. The decrease in the levels of GSH following exposure to arsenic has been found to be associated with high affinity of arsenic with GSH [51]. Chronic arsenic exposure has largely been associated with the apoptosis in the lymphocytes and involve in immunotoxic response. Viability of cells has been found to be decreased in thymus of mice following exposure to arsenic as observed in the present study suggested the immunotoxicity of arsenic. Increased production of ROS associated with enhanced lipid peroxidation and decreased levels of reduced glutathione, the activity of superoxide dismutase and catalase as observed in the present study consistent with the earlier findings [20,21]. Most of the toxic chemicals directly act on the mitochondria, disrupt its phospholipids membrane and cause mitochondrial dysfunctions [2,52,53]. Arsenic compounds have been shown to be strong inducers of apoptosis in normal and transformed cells through production of ROS [54], decreased mitochondrial membrane potential [52], activation of caspases [25,55], increased fragmentation of DNA [55], decreased expression of anti-apoptotic proteins (Bcl-2, Bcl-XL) and increased expression of pro-apoptotic proteins [55]. Martin-Chouly et al. [56] reported that inorganic arsenic directly acts on human T-cells and impaired their activity via up-regulation of several immune and stress response genes. However, the inhibition of T cell proliferation was independently of heme oxygenase −1 expression and monocyte related accessory signals [56]. Exposure to arsenic through semiconductor elements including indium arsenide and gallium arsenide could induce alterations in gene expression and immune response associated with increased production of ROS which might be involved in the apoptosis and necrosis in T-lymphocytes and neuronal cells [57,58]. A decreased in the activity of cytochrome c oxidase, mitochondrial membrane potential and increased activity of mitochondrial caspase-3, number of cells in sub G1 peak and number of apoptotic and necrotic cells following exposure to arsenic in mice as observed in the present study is consistent with the earlier reports.

Experimental studies have reported that weight of organs (thymus, spleen, adrenals) responsible for immune response was decreased following exposure to arsenic [20,59]. Subchronic low-level exposure to arsenic may affect immune responses [59]. Decrease body weight in experimental animals exposed to arsenic has been reported [59-61]. Decrease in the body weight indicates the general toxic effect of the chemical. A decrease in the body weight and thymus weight as observed in the present study could be due to immunotoxic response of arsenic that have been found to be recovered by co-treatment with amla and arsenic in mice.

Various pharmacological preparations and plant extracts are reported to have strong antioxidant potential and used against arsenic induced oxidative damage to investigate their protective efficacy [2,21,30]. Phyto constituents including flavanoids found in the plant extracts are effective as radical scavengers and inhibitors of lipid peroxidation [61-63]. Amla contains a wide variety of phenolics and its derivatives associated with its strong antioxidant potential. Amla is widely accepted immune booster among the people since it possesses multiple pharmacological and immunomodulatory properties [21,30-33]. It has been reported that amla protects against the harmful action of free radicals and exhibit its ameliorating effects in biological system [32,33]. Sharma et al. [21] reported that the antioxidant potential of amla may be due to the presence of the many phyto-constituents, which provide maximum conjugation with free radical species, thus reducing the number of free radicals available and the extent of cellular damage. They further suggested that pre and post supplementation of fruit extract of amla significantly reduce arsenic induced oxidative stress in the liver as a result serum transaminases and MDA content become lowered in the liver and also increased activity of superoxide dismutase, catalase, glutathione S transferase and serum alkaline phosphatase activities [21]. Reddy et al. [64] reported that alcohol-induced oxidative stress in plasma of rats could be ameliorated through the amla that could be due to the combined effect of phytophenols present in it. In another study, Kumar et al. [65] found that amla significantly protects against lead induced toxicity by decrease generation of free radicals in one day old male broiler chicks. Haque et al. [66] suggested that amla extract was very effective in reducing cyclophosphamide induced suppression of humoral immunity in mice. They also reported that amla extract treatment in normal animals could modulate levels of certain antioxidants of kidney and liver resulted in restoration of antioxidant enzymes in cyclophosphamide treated animals and further suggested that amla or its medicinal preparations may be useful in combination therapy in cancer patients. The supplementation with Triphala (*Terminalia chebula, Terminalia belerica and Emblica officinalis*) prevents the noise-stress induced changes in the antioxidant as well as cell-mediated immune response in rats [67]. Enhanced lipid peroxidation and decreased levels of reduced glutathione, activity of superoxide dismutase and catalase following exposure to arsenic has been found to be protected following simultaneous treatment with arsenic and amla in mice suggesting protective efficacy of amla.

In the present study, treatment with amla alone had no significant effect on cell viability, lipid peroxidation, levels of reduced glutathione, the activity of superoxide dismutase, Catalase and other apoptotic markers including the activity of caspase–3 and cytochrome c oxidase. However, decreased cell viability has been found to be protected following co-treatment with arsenic and amla in thymus of mice. Also, increased levels of lipid peroxdation, activity of caspase-3 and decreased levels of reduced glutathione, activity of superoxide dismutase, catalase and activity of cytochrome c oxidase following arsenic exposure were protected in mice co-treated with arsenic and amla. Such an immunoprotective effect of amla may attribute to its antioxidant potential to counteract free radicals and prevented from enhanced oxidative stress. The fruit extract of amla has been reported to enhance cyto-protection, decrease apoptosis and DNA fragmentation [68,69]. It has also been found to protect against heavy metals induced clastogenicity [70]. Chromium (VI) induced free radical generation associated with enhanced oxidative stress has been found to protected following treatment with amla [30]. Further, chromium (VI) induced apoptosis, DNA fragmentation and immunosuppressive effects on lymphocyte proliferation has been ameliorated following treatment with amla and it also restored the altered levels IL-2 and γ-IFN [30]. Decreased activity of cytochrome c oxidase, mitochondrial membrane potential and increased activity of mitochondrial caspase-3, number of cells in sub G1 peak and number of apoptotic and necrotic cells in arsenic exposed mice have also been found to be protected following simultaneous treatment with arsenic and amla in the present study.

## Conclusions

In conclusion, the results of the present study clearly indicated that arsenic induced free radical generation and enhance oxidative stress leading to apoptosis in thymocytes of mice. Arsenic induced decrease mitochondrial membrane potential has been found to increased following co-treatment with arsenic and amla. Further, increased number of apoptosis, necrotic cells and DNA damage following exposure to arsenic has also been found to be decreased following co-treatment with arsenic and amla indicates the anti-apoptotic property of amla that could be due to its strong antioxidative potential. Although the results of the present study exhibit immunomodulatory effects of amla through its antioxidant properties, further studies are required to understand the detailed mechanism of immunoprotection.

## Competing interests

The authors declare that they have no financial or personnel competing interests.

## Authors’ contributions

MS: designed the experiment; MS and SY conducted research and drafting of the manuscript; VS: acquisition of data; analysis and interpretation of data; statistical analysis; SK: review of the manuscript; analysis and interpretation of data; obtained funding; administrative support; study supervision. All authors read and approved the final manuscript.

## Pre-publication history

The pre-publication history for this paper can be accessed here:

http://www.biomedcentral.com/1472-6882/13/193/prepub
